# Implantation of a Dual-chamber Pacemaker in a Patient with Dextrocardia, Situs Inversus, and Sick Sinus Syndrome

**DOI:** 10.19102/icrm.2022.130201

**Published:** 2022-02-15

**Authors:** Matthew Hakimi, Maya Yabumoto, Jack Sun, Michael Rochon-Duck, David Donaldson

**Affiliations:** ^1^Department of Medicine, Division of Cardiology, University of California Irvine Medical Center, Orange, CA, USA; ^2^Department of Cardiothoracic Surgery, University of California Irvine Medical Center, Orange, CA, USA

**Keywords:** Dextrocardia, pacemaker, situs inversus

## Abstract

Situs inversus with dextrocardia is a rare congenital anomaly that presents a unique challenge for the consultant electrophysiologist. Implantation of cardiac device in these patients may be challenging owing to their individual cardiac and vascular anatomy. Consequently, adverse procedural outcomes are more common in this group and an informed pre- and intraoperative approach is critical. In this article, we present the relevant patient findings and implications for the electrophysiologist, including operative approaches. We then examine them in the context of an actual case, having implanted an intracardiac permanent pacemaker with a right-sided approach via the conventional method in a patient with dextrocardia situs inversus who had undergone multiple surgeries for structural heart disease.

## Introduction

Dextrocardia is defined as the right-sided embryologic development of the heart, with most of the cardiac mass positioned in the right hemithorax and a base-to-apex axis pointing to the right. It is a rare congenital anomaly with an incidence of 1 per 12,000 live births. Dextrocardia may occur as situs solitus (normal visceroatrial arrangement), situs inversus (“mirror image” of normal visceroatrial arrangement), or situs ambiguous (visceroatrial isomerism). In association with situs inversus, the heart is located on the right side of the chest, and while the anteroposterior relationships of the various parts of the heart are normal, their right-to-left orientation is reversed. Major intracardiac defects may exist in patients with situs inversus dextrocardia. The most common cardiac abnormalities are anomalous pulmonary veins, transposition complexes, ventricular septal defects, and atrioventricular (AV) discordance.^1,2^

## Case presentation

We present a 49-year-old woman of East Asian descent with a medical history significant for dextrocardia, situs inversus totalis, paroxysmal atrial fibrillation, and rheumatic mitral valve stenosis with prior valvuloplasty.

**Figure 1:**
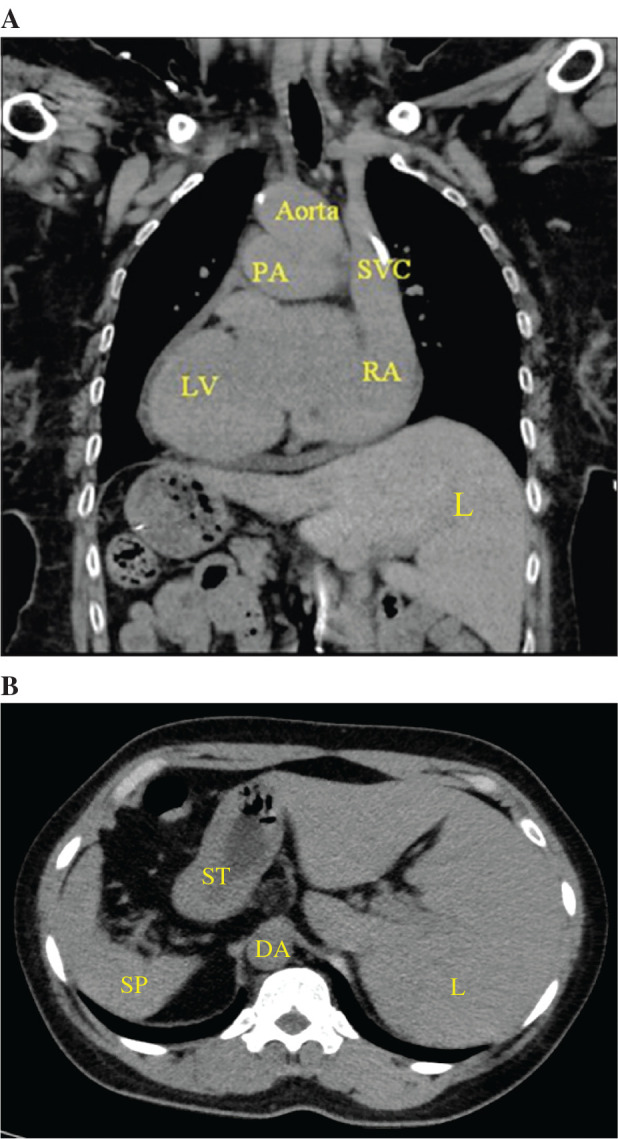
**A:** Computed tomography of the chest revealed dextrocardia with superior vena cava connected to the right atrium in the left-sided mediastinum. The aortic arch was rotated to the right-sided posterior mediastinum. **B:** Reversal of the normal anatomy in situs inversus is shown in the abdominal view. *Abbreviations*: DA, descending aorta; L, liver; LV, left ventricle; PA, pulmonary artery; SP, spleen; ST, stomach; SVC, superior vena cava.

The patient presented to our institution for a surgical evaluation of valvular heart disease following several months of progressive dyspnea on exertion. Echocardiography revealed severe calcific mitral and aortic valve stenoses, moderate tricuspid valve regurgitation, and a preserved ejection fraction. The patient was subsequently offered surgical mitral valve replacement, aortic valve replacement, tricuspid valve repair with ring annuloplasty, and bi-atrial Cox-maze and a left atrial appendage clip. In preparation for the surgeries, a computed tomography scan was performed to characterize her anatomy. A right aortic arch with a mirror-image branching pattern, left-sided liver, and stomach and spleen on the right side of the abdomen were identified **(Figures 1A and 1B)**.

**Figure 2:**
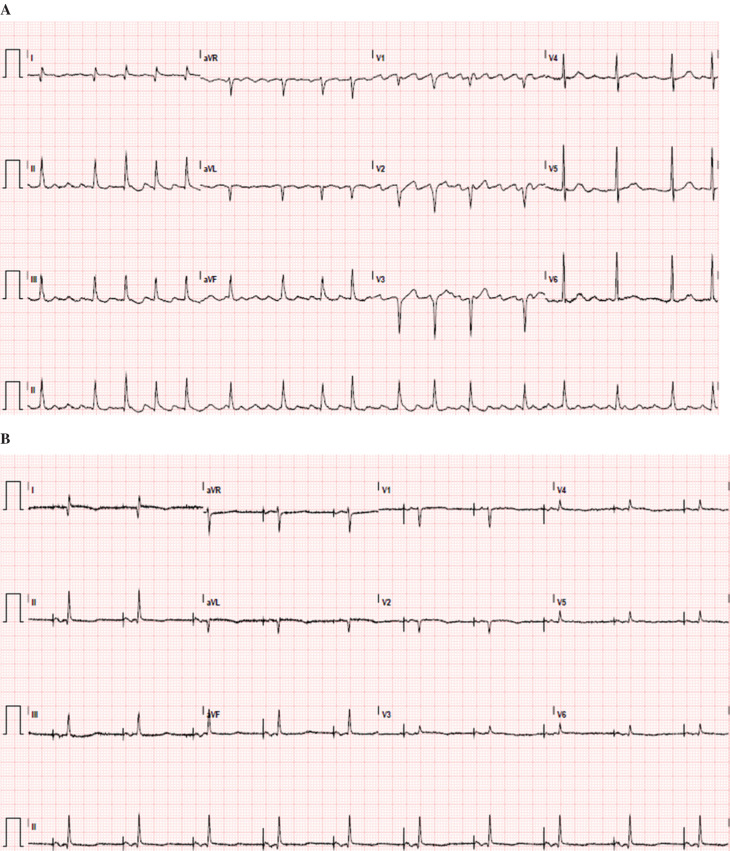
**A:** Electrocardiogram of the patient (right chest) prior to the insertion of a pacemaker. **B:** Electrocardiogram of the patient after the insertion of a pacemaker demonstrating atrial pacing.

The patient had a successful surgery and an uneventful postoperative course, yet continued to require pacing via the surgically implanted epicardial leads. The underlying rhythm was sinus and intermittent junctional bradycardia with hypotension and frequent episodes of atrial fibrillation with a difficult-to-control ventricular response **(Figure 2A)**. The patient was offered a dual-chamber permanent pacemaker (PPM) for tachycardia-bradycardia syndrome.

## Pacemaker implantation

**Figure 3:**
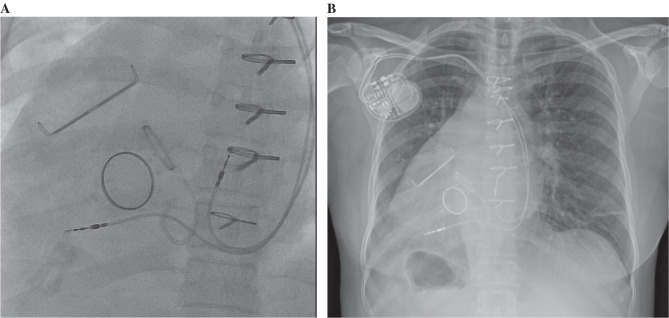
Dextrocardia with a prominent gastric bubble underneath the right hemidiaphragm is demonstrated under fluoroscopy in a 30° left anterior oblique cranial view **(A)** and chest radiograph in the anteroposterior view taken after the insertion of a dual-chamber pacemaker **(B)**.

The PPM insertion was carried out using a local anesthetic (lidocaine). A venogram was performed and bilateral intravenous lines were placed in the preoperative assessment, and venous access was gained via the right cephalic vein. Active fixation leads were implanted in the right ventricular apex (5076-52cm; Medtronic, Minneapolis, MN, USA) and the right atrial appendage (5076-45cm; Medtronic). Positioning through the right subclavian vein and into the right atrium and ventricle was confirmed with fluoroscopy. The patient tolerated the procedure well and was subsequently discharged home. Follow-up visits in our clinic have been unremarkable clinically and with regard to the device function. **Figure 3** shows the final position of the leads.

## Discussion

Situs inversus with dextrocardia presents a unique challenge for the consultant electrophysiologist. Firstly, the patient’s individual anatomy may hinder the implantation of the device. An abnormal cardiac morphology and venous orientation can result in unfamiliar lead manipulation and placement, especially in the coronary sinus cannulation phase. Anomalies of the venous vascular system, such as double or absent vena cava, are commonly associated with a mirror-image dextrocardia and may preclude a percutaneous approach.^3^ Dextrocardia can also be associated with complex cardiac malformations, such as a single ventricle, double-outlet or double-inlet ventricles, and tricuspid atresia.^4^ Consequently, detailed information on the patient’s anatomy should be obtained before the procedure. This entails knowledge of any coexisting cardiac or vascular abnormality.

Fluoroscopic images may be assessed prior to starting the procedure to appropriately plan the percutaneous approach, as was performed in our case. It is sometimes helpful to have bilateral peripheral upper-extremity venous access in case the device location must be changed. Cine angiograms serve to facilitate lead positioning in the atria and the ventricle and reveal important anatomical information regarding the orientation of the septum, morphology of the venous chambers (whether trabeculated or smooth), and the presence of a venous anomaly.^5^ This relates to the interpretation of the anatomical reference points obtained with standard fluoroscopic projections. To overcome these challenges, some operators invert the fluoroscopic image from left to right to recreate a standard levocardia orientation.^6^ Flipped image orientation with opposite angulated views, ie, right anterior oblique in place of left anterior oblique and vice versa, has been utilized in implantation. These technical challenges and the aforementioned techniques may increase the operation time and should be planned accordingly prior to the procedure. In our case, the patient underwent successful implantation of a right-sided device in standard views without flipped imaging. At last, a pre-implantation assessment with cardiac computed tomographic angiography or cardiac magnetic resonance imaging can detect congenital anatomic anomalies and provide helpful information for operative planning.

The long-term prognosis for patients with dextrocardia and situs inversus totalis after successful pacemaker implantation is favorable according to the literature.^7^ In addition, implantation of a subcutaneous implantable cardioverter-defibrillator device has been demonstrated in a large multicenter study of dextrocardiac patients with situs inversus.^8^ Catheter-based therapies for atrial fibrillation with both cryoablation and radiofrequency ablation have been performed in patients with dextrocardia and situs inversus. There have been more incidents of anomalous pulmonary vein connections; therefore, the anatomy should be carefully defined before pulmonary vein isolation is performed.^9^ Further, anatomic variation in systemic venous connections can lead to anatomic variability of the phrenic nerve such that the pacing of both the right and left phrenic nerves during the procedure is critical.^10^ There is a scarcity of large-scale meta-analyses of dysrhythmias in patients with dextrocardia; therefore, it is difficult to generalize the natural progression of disease and complications of procedures such as catheter-mediated AV nodal ablation.

## Conclusion

The implantation of cardiac devices can be safely and effectively performed in patients with dextrocardia and situs inversus. Utilization of pre- and intraprocedural techniques may optimize successful outcomes as highlighted in this case.
